# Native and exotic plants with edible fleshy fruits utilized in Patagonia and their role as sources of local functional foods

**DOI:** 10.1186/s12906-020-02952-1

**Published:** 2020-05-24

**Authors:** Melina Fernanda Chamorro, Ana Ladio

**Affiliations:** INIBIOMA, CONICET, Universidad Nacional del Comahue, Quintral, 1250-8400 San Carlos de Bariloche, Río Negro Argentina

**Keywords:** Traditional uses, herbal medicine, phytotherapy, food-medicine, Berries, Native fruits, Local use, Biocultural diversity

## Abstract

**Background:**

Traditionally part of the human diet, plants with edible fleshy fruits (PEFF) contain bioactive components that may exert physiological effects beyond nutrition, promoting human health and well-being. Focusing on their food-medicine functionality, different ways of using PEFF were studied in a cross-sectional way using two approaches: a bibliographical survey and an ethnobotanical case study in a rural community of Patagonia, Argentina.

**Methods:**

A total of 42 studies were selected for the bibliographical review. The case study was carried out with 80% of the families inhabiting the rural community of Cuyín Manzano, using free listing, interviews, and participant observation. In both cases we analyzed species richness and use patterns through the edible consensus and functional consensus indices. Local foods, ailments, medicines and drug plants were also registered.

**Results:**

The review identified 73 PEFF, the majority of which (78%) were native species, some with the highest use consensus. PEFF were used in 162 different local foods, but mainly as fresh fruit. Of the total, 42% were used in a functional way, in 54 different medicines. The principal functional native species identified in the review were *Aristotelia chilensis* and *Berberis microphylla.* In the case study 20 PEFF were in current use (50% were native), and consensus values were similar for native and exotic species. These were used in 44 different local foods, mainly as fresh fruit. Only 30% were recognized for their functional value by inhabitants (mainly as gastrointestinal and respiratory treatments). The species with the highest functional consensus were the exotic *Sambucus nigra* and *Rosa rubiginosa*, followed by the native *A. chilensis, Ribes magellanicum* and *B. microphylla*. Infusions also constituted important local functional foods.

**Conclusions:**

This survey highlights the importance of studying the different local functional foods to depict the biocultural diversity of a human society. The preparation of different beverages and herbal medicines was relevant, and would be a promising subject to investigate in the future. The living heritage of PEFF appears to have undergone hybridization processes, such that exotic species play an increasingly significant role.

## Background

A poor diet, with little consumption of fruit and vegetables, among other characteristics, is known to be strongly associated with mortality through non-communicable diseases (such as cardiovascular diseases and cancer) [1]. Extra-nutritional substances present in plants have the capacity to reduce cellular oxidative stress, thus lessening the probability of developing these illnesses [2–4]. The current challenge is to generate policies centered not just on nutrients, but on healthy foods [5]. Species with edible fruits deserve special attention not only for their value per se, but also as local foods.

The importance in this study of the variety of ways of ingesting plant-based foods is highlighted by Heinrich et al. [6]. They developed the concept of “local foods” through the study of the Mediterranean diet, distinguishing those recipes that are shared in a territory or culture, and which form a fundamental part of local food knowledge [6]. This represents an interesting field of study in the subject of functional foods, a concept that originated in Japan in the 1980s as Foods for Specified Health Use (FOSHU). At the present time, “Functional Food Science in Europe” (FUFOSE) proposes that functional food is that which is “satisfactorily demonstrated to affect beneficially one or more target functions in the body, beyond adequate nutritional effects, in a way that is relevant to either an improved state of health and wellbeing and/or reduction of risk of disease” [6].

From an ethnobotanical perspective, other concepts arise such as “Folk functional foods” [7], or what we will call here “local functional foods”, based on the multifunctional character of food use by local inhabitants [8]. The study of plant-based local functional foods currently offers an opportunity for ethnopharmacological research, as a wide range of biocompounds is revealed, which is made available to humans in the different recipes used, capable of transforming human gastronomic habits.

The study of native and exotic edible plant use in rural and indigenous communities offers an opportunity to appreciate how humans have experimented with the diversity of their plant surroundings in search of wellbeing. As established by Etkin and Ross [9], plant studies in these communities should be approached with understanding of the integral nature of food and health concepts. In their search for this functionality, humans have intentionally selected dual-purpose resources (edible-medicinal) and have also tended towards diversification of food types and new forms of consumption. Consequently, we are interested in showing the multidimensionality and multifunctionality of the PEFF, considering all their plant parts in an integral way.

The study of the potential of Patagonian native fruit species is relatively recent. Progress has been made from the perspectives of agronomy [10, 11], phytochemistry [12–14] and nutrition [15, 16]. Several studies have demonstrated the antioxidant properties of some Patagonian fruits and their usefulness in regulating glucose metabolism, thus indicating their nutraceutical value [17–19]. Nevertheless, the diversity of existing species and their local foods has been little described up to now [20].

One aspect highlighted in the ethnobotanical studies carried out in Patagonia is the high proportion of exotic edible plant use in some local communities [21–23]. For example, in rural areas, the gathering of edible plants seems to be linked to environmental changes, with the incorporation of more exotic fruit-bearing species that grow wild, including invasive plants like sweet briar, elmleaf blackberry and apple (*Rosa rubiginosa* L., *Rubus ulmifolius* Schott, *Malus domestica* Borkh) [21]. Due to processes of colonization and imposition, since the arrival of Europeans the region has tended towards the cultivation of exotic fruits [24]. In market gardens and fruit farms 90% of the fruit grown is exotic in origin, such as plums and apples [25]. This pattern, called the hybridization process [26], is prevalent and of great interest when considering local foods.

In this study we focus on native and exotic plants with edible fleshy fruits (PEFF) that grow in Patagonia; that is, species that may be wild, cultivated or in an intermediate state of domestication, which bear fruit that is distinguished by its flavor, preferably sweet, and its use principally as a food resource. In general, they have a high proportion of water and sugars that are easily digested and absorbed, and they have long contributed fiber, minerals and vitamins to the diet of local populations [27]. Very little information is available concerning the total number of species involved in this group and how people have used them. In addition, far fewer studies exist that deal with the safety aspect of consumption of PEFF and their different preparations.

Understanding the use patterns of PEFF as part of the local biocultural heritage of Patagonia is crucial [27]. In recent times, as in the rest of the world, rural and urban societies of this region have experienced sociocultural and environmental changes. These changes have led to a reduction in traditional plant use [28], and some elements of the flora seem to be in danger [29]. These alterations will have serious long-term repercussions for PEFF use, as motivation for the generational transmission processes (oral traditions) is weakened, and consequently, the patrimony for future generations is reduced.

In this study we propose a cross-sectional approach which enables exotic and native species richness, local foods, medicines and their use patterns to be evaluated, and which will also help us understand in greater depth, from an ethnobotanical perspective, that diet and health are linked concepts. We will carry out a bibliographical review and a case study of a current rural community in order to synthesize existing information on the functionality of native and exotic PEFF, and analyze their use patterns and potential.

Our main questions were: Which native and exotic plants from Patagonia constitute PEFF? Which species are currently in use? What proportion is used as functional food? How many local foods are involved? This analysis of mixed information also enables us to show the continuity or change in use of functional and non-functional PEFF, shedding light on the existing biological and cultural diversity, and its potential.

## Methods

### Ethnohistorical and ethnobotanical bibliographical review

The bibliographical review involved quali-quantitative analysis of ethnohistorical and ethnobotanical texts published since 1947 which mention the use of native and exotic fruits in the Patagonian region [30]. The review was conducted using Scopus, Science Direct and Scielo, and Google Scholar search engine, as well as the library and database of the Grupo de Etnobiología. The key words and phrases used in the search were: native and exotic edible fruits and Patagonia; edible plants and Patagonia; medicinal plants and Patagonia; Patagonian flora and uses; ethnobotany of Patagonia; medicinal and alimentary uses and Patagonia, and native fruits of Patagonia. The search was carried out in Spanish and English, and a total of 2133 documents were examined. The works selected were mainly primary field studies, but compilations were also included given that each review used independent protocols for species evaluation, according to the author of the work.

### Study region

Location of Database publications: The articles selected were from studies carried out in Argentine rural and urban communities with non-indigenous people, Creoles, and people of Mapuche-Tehuelche-Selk’nam ancestry, distributed over an area extending approximately between 37^o^ and 54^o^ lat. S. Of the articles studied, 58% dealt with organized indigenous communities, while the remainder were rural and/or urban communities which were pluricultural in character. The phytogeographical heterogeneity of the bibliographical approach led to the inclusion of a high diversity of plants, life forms and botanical families that were used by the majority of the indigenous populations in the region [31, 32].

Fieldwork location: The fieldwork was carried out in the rural community of Cuyín Manzano, which is located in Nahuel Huapi National Park, (40° 45 S and 71° 10 W), 70 km from Bariloche city. This location lies within the Andino Norpatagonica Biosphere Reserve (UNESCO). The climate in the region is temperate-cold and humid, with a Mediterranean precipitation regime, such that rain and snow fall principally in winter (MAT 7.2 °C). The rural community is located in an ecotonal environment between steppe and forests of *Austrocedrus chilensis* and *Nothofagus* spp. The population is of mestizo origin; some individuals are direct descendants of the Mapuche people, while others have mixed ancestry, known locally as *Criollos* (Creoles). At present only 13 families live here, due to strong processes of emigration. The economic activity of inhabitants has become more varied with time; they work with livestock, tourism, handicrafts and also as employees in the state school hostel and a private ranch. This community has a long-standing history of settlement in the area, according to archeological records [33]. Our previous studies in this location [34–36] have shown that this population is very representative of a typical Patagonian rural community. Considering its low population density at present, the native and mixed ancestry of the people, its immigration and migration process, its contact with the city and the maintenance of its traditions, Cuyin Manzano reflects shared sociocultural patterns with other rural settlements. In addition, the fieldwork was greatly facilitated due to the close ties of trust we have built up with the people over several years.

### Database design

The information obtained from each source was systematized in a database according to the following criteria: 1) The species should be identified taxonomically in the original study; 2) It should grow in Argentine Patagonia; 3) Native and exotic species were included. Native species were defined here as plants whose origin is central or southern Argentina, below 35° southern latitude, and exotic species were those which did not fulfill this condition [31]; 4) Wild, semi-domesticated and cultivated species were included; 5) The species were categorized as 1 exclusively edible, and 2, functional, when the plant had at least one functional citation (edible, with also one or more medicinal uses) in the bibliography. Following these criteria, 42 studies and 358 citations were included. Each citation corresponds to a species referred to in a publication, with its corresponding ethnobotanical information.

The following information was selected: species, common and indigenous names, richness of local foods, medicinal uses, herbal medicines made with PEFF and parts of the plant used (named plant drugs). The local foods were classified as: fresh fruit (F), jams and sweets (S), non-alcoholic drinks (D), fermented drink called “chicha” (Ch), other (OT). The classification of ailments was adapted from Molares and Ladio [37]: respiratory (RS), gastrointestinal (GI), urinary (U), pain and inflammation (PI), dermatologic (DE), fever (F), obstetric (OBS), gynecological (GYN), blood (BL), circulatory and heart (CH), nervous system (NS), cultural syndromes (CS), osteo-articular and muscular (OA), allergies (AL), dentistry (DEN), ophthalmological (OP), refreshment (RE), others (OT). The different herbal medicines were classified according to method of administration: fruit ingestion (F), infusion (In), decoction (De), bathing, washing and rubbing (Ba), gargling (Ga), poultices and compresses (Ca), creams and ointments (Oi), other (Ot). The plant drugs were categorized according to the part of the plant used: fruit (F), flowers (Fl), leaves (L), branches (B), bark or stalk (S), root (R), the whole plant (Pl).

### Field study

Fieldwork was carried out according to the Code of Ethics of the International Society of Ethnobiology (ISE) and the Consensus Statement on Ethnopharmacological Field Studies [38]. Free listing, semi-structured interviews, in-depth interviews and participant observation were performed with participants [39, 40], prior oral informal consent. If the participant consented to being recorded during the interview, oral consent was also tape-recorded. This procedure is supported by national research regulatory agencies and international agreements (Argentinian National Law N.°27,246). A total of 80% of the population (11 households) took part in the study, including several members of each family. The ages of the adult participants ranged from 31 to 67 years. Inhabitants were consulted on their knowledge of the PEFF that grew in the area, and their alimentary and functional uses. The information gathered was reorganized following the categories used for the bibliographical review. The database included 125 citations with information on PEFF. Each citation corresponds to a species referred by each interviewee.

### Data analysis

The same quali-quantitative analysis [41] was performed for both approaches. Due to the categorical nature of the data, the analyses were principally non-parametric, using the SPSS 23 package for Windows.

### Species richness

Species richness (S) was calculated in total, according to biogeographical origin, and by botanical family. Native and exotic species richness were compared using the binomial test (*p* < 0.05) [42].

### Consensus index

Whether a native or exotic species was used as edible or functional was evaluated by consensus indices, mainly utilized for use pattern analysis of ethnobotanical data [43–46]. These indices enable us to consider the agreement in a community or between diverse authors (and their publications) on the use of a certain species or plant family. The cultural importance index of edible plants (CIE) was calculated for the species and for the families: CIE = FC/N × 100, where FC = the frequency of citation of the species or family and *N* = the number of total publications/informants.

We also calculated the CIF index for functional species and families, as CIF = FC/N × 100, where FC = the frequency of citation of the functional species or family. The Mann Whitney test was performed to compare these indexes for native and exotic species (*p* < 0.05).

### Local foods

The percentage of each category was calculated in relation to the total reported methods of use of all the PEFF, that is, the total foods. %SC _**A**_ = S_**SCA**_/ƩS_**SCA**.**.i**_ × 100. Where %SC_**A**_ = the percentage of the subcategory A (for instance, drinks); S_**SCA**_ = the richness of species of the subcategory A; and ƩS_**SCA**.**.**i_ = the total of local foods.

### Use value

The use value is an index that indicates the versatility of a species, that is, the diversity of ailments that can be treated by a given species [37, 47, 48]. It was obtained as follows: UVs = ƩUVis/N [49], where UVis = the number of different medicinal uses registered by publication/informant for species S.

### Herbal medicines and plant drugs

Similarly to local foods, the percentage of each category was calculated in relation to the sum of the plant richness for each subcategory. That is, in relation to the total medicines or plant drugs (medicinal parts of the plants).

### Species identification

In the field study all native and exotic plant species were mentioned by their common names, and were later identified taxonomically by the authors. The collection of wild and cultivated species with fleshy fruits was performed with the assistance of local dwellers. In addition, field herbaria and photographs were utilized in the interviews to confirm the taxonomic identity of plants. Plant identification followed Correa [50–52] Plant specimens were placed in the Ecotono-Ethnobiology Group-INIBIOMA- University of Comahue herbarium, and will be deposited in the Herbarium of the Centro Regional Universitario Bariloche (BCRU). Voucher specimens of all reported species are shown in Table 3. It should be noted that the Berberidaceae family was treated according to Landrum [53]. All scientific names were updated using World Flora Online (www.worldfloraonline.org).

## Results

### Bibliographical study

#### Richness and use pattern (CIE) of PEFF

**Fig. 1:**
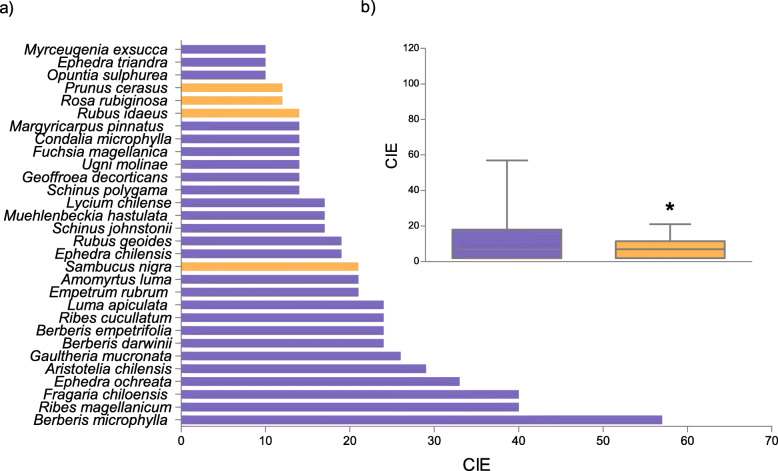
Edible Consensus Index (CIE) from the review analyses **a**) The 30 most cited PEFF from Patagonia (Argentina) **b**) Comparison between native and exotic species. Blue = native; orange = exotic. Asterisks illustrate significant difference, (*p* < 0,05 Mann Whitney test)

A total richness (S) of 73 species was found through the bibliographical review (Table 1, Fig. 1a). The average richness per study was 8.5 species (min: 1, max: 43). Richness of native species (78%, 57 species) was notably greater than that of exotic plants (22%, 16 species) (*p* < 0.05, binomial test). Coincidentally, the CIE of native PEFF was higher than for exotic plants (*p* < 0.05, Mann Whitney test) (Fig. 1b).

**Table 1:** PEFF from Patagonia (Argentina) and their local foods (*N* = 42)

Species	Local name	Family	Origin	F	S	D	Ch	O	Total
*Amomyrtus luma* (Molina) D. Legrand & Kausel	luma	Myrtaceae	na	1	1	1	1	1	5
*Aristotelia chilensis* (Molina) Stuntz	maqui	Elaeocarpaceae	na	1	1	1	1	1	5
*Austrocactus patagonicus* (F.A.C.Weber ex Speg.) Hosseus	chupasangre	Cactaceae	na	1	0	0	0	0	1
*Berberis darwinii* Hook.	michay	Berberidaceae	na	1	1	1	1	1	5
*Berberis empetrifolia* Lam.	calafate, calafatillo	Berberidaceae	na	1	1	1	1	0	4
*Berberis ilicifolia* L.f.	calafate	Berberidaceae	na	1	0	1	0	0	2
*Berberis microphylla* G.Forst.	calafate	Berberidaceae	na	1	1	1	1	1	5
*Berberis ruscifolia* Lam.	quebrachillo	Berberidaceae	na	1	0	0	0	0	1
*Berberis serratodentata* Lechl.	saloll	Berberidaceae	na	1	0	0	0	0	1
*Berberis trigona* Kunze ex Poepp. & Endl.	michay, calafate	Berberidaceae	na	1	1	0	1	0	3
*Cereus aethiops* Haw. *	albaricoque	Cactaceae	na	1	0	0	0	0	1
*Condalia microphylla* Cav.	piquillín	Rhamnaceae	na	1	1	1	0	1	4
*Empetrum rubrum* Vahl ex Willd.	mutilla	Ericaceae	na	1	0	0	0	0	1
*Ephedra americana* Humb. & Bonpl. ex Willd. *	Nm	Ephedraceae	na	1	0	1	0	0	2
*Ephedra chilensis* C. Presl *	sulupe, solupe, pingo-pingo	Ephedraceae	na	1	0	1	0	0	2
*Ephedra ochreata* Miers	sulupe, solupe	Ephedraceae	na	1	0	1	1	1	4
*Ephedra triandra* Tul.	tramontana	Ephedraceae	na	1	1	1	0	0	3
*Fragaria chiloensis* (L.) Mill.	frutilla	Rosaceae	na	1	1	1	1	1	5
*Fragaria vesca* L.	frutilla	Rosaceae	ex	1	0	0	0	0	1
*Fuchsia magellanica* Lam.	chilco	Onagraceae	na	1	0	0	0	0	1
*Gaultheria antarctica* Hook.f.	Nm	Ericaceae	na	1	0	0	0	0	1
*Gaultheria mucronata* (L.f.) Hook. & Arn.	chaura	Ericaceae	na	1	0	0	1	0	2
*Gaultheria phillyreifolia* (Pers.) Sleumer	chaura	Ericaceae	na	1	0	0	0	0	1
*Gaultheria poeppigii* DC.	manzanita	Ericaceae	na	1	1	0	0	1	3
*Gaultheria pumila* (L.f.) D.J.Middleton	mutilla	Ericaceae	na	1	0	0	0	0	1
*Geoffroea decorticans* (Hook. & Arn.) Burkart *	chañar	Fabaceae	na	1	1	1	1	1	5
*Gunnera magellanica* Lam.	Nm	Gunneraceae	na	1	0	0	0	0	1
*Luma apiculata* (DC.) Burret *	arrayán	Myrtaceae	na	1	1	1	1	1	5
*Luzuriaga radicans* Ruiz & Pav.	quillineja, quila del monte	Alstroemeriaceae	na	0	0	0	0	0	0
*Lycium chilense* Bertero	yaoyín	Solanaceae	na	1	0	0	0	0	1
*Lycium tenuispinosum* Miers	yaoyín	Solanaceae	na	1	0	0	0	0	1
*Maihuenia patagonica* (Phil.) Britton & Rose *	chupasangre	Cactaceae	na	1	0	0	0	0	1
*Maihuenia poeppigii* (Otto ex Pfeiff.) F.A.C.Weber *	maihuén	Cactaceae	na	1	0	0	0	0	1
*Maihueniopsis darwinii* (Hensl.) F. Ritter *	Nm	Cactaceae	na	1	0	0	0	1	2
*Margyricarpus pinnatus* (Lam.) Kuntze	perlilla, yerba de la perdíz	Rosaceae	na	1	0	0	0	0	1
*Morus alba* L.	mora	Moraceae	ex	1	0	0	0	0	1
*Morus nigra* L.	mora	Moraceae	ex	1	0	0	0	0	1
*Muehlenbeckia hastulata* (Sm.) I.M.Johnst.	quilo, quineo, zarzaparrilla	Polygonaceae	na	1	1	1	0	1	4
*Myrceugenia exsucca* (DC.) O.Berg	pitra, patagua	Myrtaceae	na	1	0	0	0	1	2
*Myrteola nummularia* (Lam.) O. Berg *	murta	Myrtaceae	na	1	0	1	0	1	3
*Opuntia sulphurea* Gillies ex Salm-Dyck *	penca	Cactaceae	na	1	0	0	1	0	2
*Persea lingue* (Miers ex Bertero) Nees **	lingue	Lauraceae	na	0	0	0	1	0	1
*Philesia magellanica* J.F.Gmel.	coicopihue	Philesiaceae	na	1	0	0	0	0	1
*Podocarpus nubigenus* Lindl.**	mañío macho	Podocarpaceae	na	1	0	0	0	1	2
*Prumnopitys andina* (Poepp. ex Endl.) de Laub.***	lleuque	Podocarpaceae	na	1	0	0	1	1	3
*Prunus armeniaca* L. ****	damasco	Rosaceae	ex	1	0	0	0	0	1
*Prunus avium* (L.) L. *	cerezo	Rosaceae	ex	1	0	0	0	0	1
*Prunus cerasus* L.	guindo	Rosaceae	ex	1	0	0	0	0	1
*Prunus domestica* L.	ciruelo	Rosaceae	ex	1	0	0	0	0	1
*Ribes aureum* Pursh	corinto	Grossulariaceae	ex	1	0	0	0	0	1
*Ribes cucullatum* Hook. & Arn.	parrilla, zarzaparrilla	Grossulariaceae	na	1	1	0	1	1	4
*Ribes densiflorum* Phil.	grosellero	Grossulariaceae	na	1	0	0	0	0	1
*Ribes magellanicum* Poir.	parrilla, zarzaparrilla	Grossulariaceae	na	1	1	0	1	1	4
*Ribes nigrum* L.	grosellla	Grossulariaceae	ex	1	0	0	0	0	1
*Ribes punctatum* Ruiz & Pav.	grosella	Grossulariaceae	na	1	0	0	0	0	1
*Ribes rubrum* L.	corinto	Grossulariaceae	ex	1	0	0	0	0	1
*Ribes uva-crispa* L.	grosella	Grossulariaceae	ex	1	0	0	0	0	1
*Rosa rubiginosa* L.	rosa mosqueta	Rosaceae	ex	1	1	1	0	0	3
*Rubus geoides* Sm.	frutilla de la cordillera	Rosaceae	na	1	1	1	0	1	4
*Rubus idaeus* L.	frambuesa	Rosaceae	ex	1	1	0	0	0	2
*Rubus radicans* Cav.	miñe-miñe	Rosaceae	na	1	0	0	0	1	2
*Rubus ulmifolius* Schott	murra	Rosaceae	ex	1	1	0	0	0	2
*Sambucus nigra* L. *	sauco	Adoxaceae	ex	1	1	1	0	1	4
*Schinus fasciculata* (Griseb.) I.M. Johnst.	molle	Anacardiaceae	na	1	0	1	1	0	3
*Schinus johnstonii* F.A. Barkley	molle	Anacardiaceae	na	1	0	1	1	1	4
*Schinus molle* L.	aguaribay	Anacardiaceae	na	1	1	1	0	1	4
*Schinus odonellii* F.A.Barkley	molle	Anacardiaceae	na	1	0	1	0	0	2
*Schinus patagonicus* (Phil.) I.M. Johnst.	laura	Anacardiaceae	na	1	0	0	1	0	2
*Schinus polygama* (Cav.) Cabrera *	molle	Anacardiaceae	na	1	0	1	1	1	4
*Schinus roigii* Ruiz Leal & Cabrera	molle	Anacardiaceae	na	0	0	0	0	0	0
*Tristerix corymbosus* (L.) Kuijt	quintral	Loranthaceae	na	1	0	0	0	0	1
*Ugni molinae* Turcz.	uñi, murta	Myrtaceae	na	1	1	1	0	1	4
*Vitis vinifera* L.*	parra	Vitaceae	ex	1	0	0	0	0	1
Total				70	22	25	20	25	162

The asterisks show the species listed in the IUCN Red List of Threatened Species * = Least concern, ** = Near threatened, *** = Vulnerable, **** = Endangered, *na* native, *ex* exotic, *F* fruit, *S* sweets, *D* drinks, *Ch* chicha, *O* Others, *Nm* not mentioned

The native species with highest CIE were *Berberis microphylla, Ribes magellanicum*, *Fragaria chiloensis*, *Ephedra ochreata*, *Aristotelia chilensis*, *Gaultheria mucronata*, *Berberis darwinii* and *Berberis empetrifolia* (Fig. 1a). The exotic species with highest CIE were *Sambucus nigra*, *Rubus idaeus*, *Prunus cerasus*, *Rosa rubiginosa*, *Ribes aureum* and *Ribes uva-crispa*.

The PEFF belonged to 24 botanical families, the main ones being Rosaceae (12 species), Grossulariaceae (8 species), Anarcardiaceae and Berberidaceae (7 species each). The families with the highest CIE values were Berberidaceae, Rosaceae and Grossulariaceae.

#### Local foods with PEFF

In the bibliography, 162 local foods were identified. Most publications reported the use of fresh fruits (43% of the total local foods). A wide variety of preparations were found that generally included the addition of sugar, such as jams and jellies (14%). The most significant species used to make jams were *B. microphylla*, *B. darwinii*, *R. magellanicum*, *R. cucullatum* and *F. chiloensis*.

Another important way of taking advantage of PEFF was the preparation of drinks, mainly as soft drinks. These included non-alcoholic beverages (16%) made from the fruit of 25 different species, such as *Ephedra triandra*, *Aristotelia chilensis* and *Berberis microphylla*. The next most common drink was the fermented “chicha” (12%), which used 20 different PEFF, amongst which stood out *Schinus polygama, Geoffroea decorticans, R. magellanicum* and *Amomyrtus luma.* The production of “chicha” from *A. luma* mixed with fruits of other species is also mentioned*.*

Fifteen percent of local foods includes preparations with cooked, boiled or roasted fruits, as in the case of *Maihueniopsis darwinii*. Wines are also prepared, such as those made from *A. chiloensis* fruit, or liqueurs from *B. microphylla* or *Gaultheria poeppigi* fruits. Similarly, this category includes flours such as those made with *Condalia microphylla* and *Geoffroea decorticans* fruits.

#### Richness and use pattern (CIF) of functional PEFF

Of the total number of species registered, the proportion of functional PEFF was 42% (31 species, Table 2), similar to the non-functional species (58%, 42 species) (*p* > 0.05, binomial test). The richness of functional species was composed mainly of native species (90%, *p* < 0.05, binomial test). It was also found that the CIF of native species was significantly higher than that of exotic plants (*p* < 0.05, Mann Whitney test).

**Table 2:** Functionals PEFF from Patagonia (Argentina)

Specie	CIF	UV	Ailments	Medicines and used part
*Aristotelia* chilensis (Molina) Stuntz	19,0	0,55	GI, F, RS, DE, PI, U, NS, OA y M, S, R, OT	F-In(l, f)-Ca(l)-Ba(f, l)-Ga(f, l)
*Sambucus nigra* L.	14,3	0,31	RS, F, GI, PI, CS, OT	In(f, fl)-De(l, ba, f)-Ba
*Ephedra ochreata* Miers	14,3	0,24	GI, DE, OA y M, U, GIN, PI, RS	In(f)-De (br, ro)-Ga (st)
*Ribes magellanicum* Poir*.*	14,3	0,19	BL, GI, RS, CH, F, AL	In(l, br, ba)-De (ro)
*Berberis microphylla* G.Forst*.*	11,9	0,38	F, GI, OT, RS, DE, PI	F-In(f, l)-De (pl, ba, ro)
*Amomyrtus luma* (Molina) D. Legrand & Kausel	11,9	0,17	PI, NS, CS, GI, OT	In (br, ba, l)-De (br, ba)-Nm(f, pl)
*Luma apiculata* (DC.) Burret	11,9	0,36	GI, DE, RS, NS, PI, F BL, OT	F-In(f, fl, l, ba)- De(l, ro, ba)-Ba(l)-Ex (ba)-Gar(l)
*Fragaria chiloensis* (L.) Mill.	11,9	0,26	OBS, GIN, GI, BL	In(l, pl)-De (ro, pl)
*Schinus johnstonii* F.A. Barkley	9,5	0,10	OD, PI, RS	In (st)-De (ro)-Ex(l)
*Geoffroea decorticans* (Hook. & Arn.) Burkart	9,5	0,12	RS, BL	F-In(f, fl, l, ba)-De (ba)-Ot (ba)
*Ribes cucullatum* Hook. & Arn.	9,5	0,17	GI,BL, U, GIN, CH	In(l, ba)-De (ro)
*Berberis darwinii* Hook.	7,1	0,14	F, GI, PI, OT	In(f, l)-De (ro)
*Berberis empetrifolia* Lam.	7,1	0,05	GI, F	F-De (ro)
*Ephedra chilensis* C.Presl	7,1	0,17	U, GIN, GI, DE, OA y M	In(f, br)-De (br, ro, pl)
*Fuchsia magellanica* Lam.	7,1	0,26	U, GIN, CH, F, R, GI	In(l, fl, ba), De (ba, l, fl)
*Margyricarpus pinnatus* (Lam.) Kuntze	7,1	0,05	U, GI	In(f)-De (pl)
*Schinus odonellii* F.A.Barkley	4,8	0,02	NS	Nm(l)
*Ephedra triandra* Tul.	4,8	0,00	Nm	Nm (br)
*Myrceugenia exsucca* (DC.) O.Berg	4,8	0,05	DE, OA Y M	Nm (l)
*Muehlenbeckia hastulata* (Sm.) I.M.Johnst.	4,8	0,12	GI, U, DE, CH, OA y M	In(l)-Oi (ro)
*Condalia microphylla* Cav.	4,8	0,05	GI, F	De (ba)
*Rosa rubiginosa* L.	4,8	0,12	RS, PI, DE, OD	De(f)
*Schinus fasciculata* (Griseb.) I.M. Johnst.	2,4	0,10	PI, RS, DE, OA y M	De (br)-Ba (br)
*Schinus molle* L.	2,4	0,02	RS	De (ba, ro)
*Schinus polygama* (Cav.) Cabrera	2,4	0,00	Nm	Nm
*Schinus roigii* Ruiz Leal & Cabrera	2,4	0,00	Nm	Nm
*Berberis trigona* Kunze ex Poepp. & Endl*.*	2,4	0,02	F	F
*Ephedra americana* Humb. & Bonpl. ex Willd.	2,4	0,12	GI, U, GIN, DE, OA y M	In(f)-De (br, ro)
*Gaultheria phillyreifolia* (Pers.) Sleumer	2,4	0,05	DE, F	Nm
*Tristerix corymbosus* (L.) Kuijt	2,4	0,07	GI, RS, OF	F-Nm(l, pl)
*Prunus cerasus* L.	2,4	0,12	GI, U, RS, CH, NS	Nm

*RS* respiratory, *GI* gastrointestinal, *UI* urinary, *PI* pain and inflammation, *DE* dermatologic, *F* fever, *OBS* obstetric, *GIN* gynecological, *BL* blood, *CH* circulatory and heart, *NS* nervous, *CS* cultural syndromes, *OA y M* osteo-articular and muscular, *AL* allergies, *DEN* dentistry, *OP* ophthalmologic, *RE* refreshment, *OT* others, *F* fruit ingestion, *In* infusion, *De* decoction, *Ba* bath, *Ga* gargle, *Ex* exudate, *Oi* ointment, *f* fruit, *l* leaf, *fl* flower, *br* branch, *ro* roots, *ba* bark, *pl* whole plant, *st* steam, *Nm* not mentioned

The principal functional species according to the CIF values were the native *Aristotelia chilensis*, *Ribes magellanicum, Ephedra ochreata, Berberismicrophylla*, *Fragaria chiloensis*, *Luma apiculata* and *Amomyrtus luma*, and the exotic *Sambucus nigra*, *Rosa rubiginosa* and *Prunus cerasus* (Table 2).

The functional PEFF found belonged to 14 botanical families. Among these were Anacardiaceae (6 species); Berberidaceae, Ephedraceae and Rosaceae with 4 species each, and Myrtaceae with 3 species. At botanical family level, Elaeocarpaceae, Ephedraceae, Grossulariaceae, Myrtaceae, Anacardiaceae, Rosaceae, Adoxaceae and Berberidaceae were families with high CIF values.

The functional PEFF registered in the bibliography were used for a wide range of ailments (Table 2), mainly gastrointestinal, respiratory, dermatological, gynecological, obstetric, related to the nervous system, heart and circulatory system, fever, pain and inflammation. The most versatile species according to their UV value were *Aristotelia chilensis,* used to treat more than ten ailments, followed by *Berberis microphylla, Luma apiculata, Sambucus nigra, Fragaria chiloensis* and *Fuchsia magellanica* (Table 2).

#### Medicines with PEFF

The PEFF used as herbal medicines totaled 54 (Table 2), which mainly included forms that can be ingested, but also some of external use. The most important were two forms of drink: decoctions (35%) and infusions (33%). For example, infusions made of leaves or bark of *R. magellanicum* were used to “componer la sangre” (depurative) and for digestive ailments. A decoction of *B. microphylla* bark is also used to bring down a fever, or its fruit is used to combat diarrhea. The direct ingestion of *A. chilensis* fruit was also used to treat fever and diarrhea.

Among the methods of external use that stand out were: bathing (9%), for example, using *Luma apiculata* leaves to bathe infected wounds; gargling (6%), with the same species but in this case to treat lesions of the gums. The application of exudates and poultices (4% each) has also been documented, for which the aerial parts of the plant were generally used, such as the exudate of *Schinus johnstonii* leaves used to treat toothache. The exudate of *L. apiculata* as an anti-inflammatory is also frequently cited in the literature [21, 54, 55]. Poultices such as those prepared from dried *A. chilensis* leaves were used to clean wounds. Creams and fresh fruit made up the remaining 9%.

The parts of the plant most frequently used medicinally were the leaves (22%), fruits (19%), bark or stalk (18%) and roots (18%).

### Fieldwork

#### Richness and use pattern (CIE) of PEFF

**Fig. 2:**
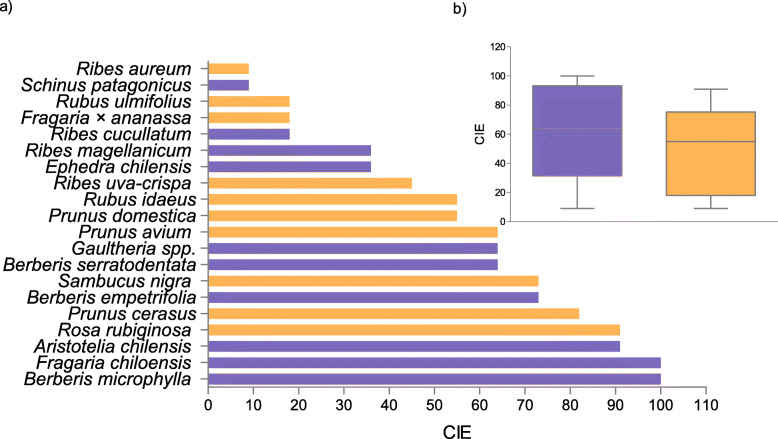
Edible Consensus Index (CIE) from Cuyín Manzano **a**) The 20 cited PEFF **b**) Comparison between native and exotic species. Blue = native; orange = exotic. No significant diference was found (*p* < 0,05 Mann Whitney test)

A total richness of 20 PEFF was found (Table 3, Fig. 2a), an average of 11 species being cited per informant (min: 7, max: 19). This richness was divided equally between native (10 species) and exotic (10 species) plants (p > 0.05, binomial test). In contrast to the findings from the review, in Cuyín Manzano the CIE was the same for native and exotic species (p > 0.05, Mann Whitney test) (Fig. 2b).

**Table 3:** PEFF from Cuyín Manzano and their local foods (*N* = 11)

Specie	Voucher no.	Local name	Family	Origin	F	S	D	O	Total
*Aristotelia chilensis* (Molina) Stuntz	500 MC	maqui	Elaeocarpaceae	na	1	0	0	0	1
*Berberis empetrifolia* Lam.	501 MC	michay de la costa	Berberidaceae	na	1	0	0	0	1
*Berberis microphylla* G.Forst.	284 MC	michay, calafate	Berberidaceae	na	1	1	1	1	4
*Berberis serratodentata* Lechl.	528 MC	michay de la cordillera	Berberidaceae	na	1	0	0	0	1
*Ephedra chilensis* C.Presl	503 MC	cola de caballo	Ephedraceae	na	1	0	0	0	1
*Fragaria × ananassa* (Duchesne ex Weston) Duchesne ex Rozier	504 MC	futilla cultivada	Rosaceae	ex	1	1	0	1	3
*Fragaria chiloensis* (L.) Mill.	504 MC	frutilla de campo	Rosaceae	na	1	0	0	0	1
*Gaultheria mucronata* (L.f.) Hook. & Arn.	505 MC	mutilla	Ericaceae	na	1	0	0	0	1
*Prunus avium* (L.) L. *	506 MC	cerezo	Rosaceae	ex	1	1	0	1	3
*Prunus cerasus* L.	507 MC	guindo	Rosaceae	ex	1	1	1	1	4
*Prunus domestica* L.	507 MC	ciruelo	Rosaceae	ex	1	1	0	1	3
*Ribes aureum* Pursh	BCRUE113	parrilla cultivada	Grossulariaceae	ex	1	1	0	1	3
*Ribes cucullatum* Hook. & Arn.	510 MC	parrilla, zarzaparrilla	Grossulariaceae	na	1	0	0	0	1
*Ribes magellanicum* Poir.	511 MC	zarzaparrilla, parrilla de campo	Grossulariaceae	na	1	0	0	0	1
*Ribes uva-crispa* L.	BCRUE114	grosella	Grossulariaceae	ex	1	1	0	1	3
*Rosa rubiginosa* L.	Ladio 136	mosqueta	Rosaceae	ex	1	1	1	1	4
*Rubus idaeus* L.	Ladio 154	frambuesa	Rosaceae	ex	1	1	1	1	4
*Rubus ulmifolius* Schott	Ladio 137	murra	Rosaceae	ex	1	0	0	1	2
*Sambucus nigra* L. *	516 MC	saúco	Adoxaceae	ex	1	1	0	0	2
*Schinus patagonicus* (Phil.) I.M. Johnst*.*	Ladio 104	laura	Anacardiaceae	na	1	0	0	0	1
Total					20	10	4	10	44

The asterisks show the species listed in the IUCN Red List of Threatened Species * = Least concern. *na* native, *ex* exotic, *F* fruit, *S* sweets, *D* drinks, *O* others

The native species with highest CIE were *Berberis microphylla*, *Fragaria chiloensis* and *Aristotelia chilensis* (Fig. 2a). These were followed by other Berberidaceae species, such as the “michay de la costa” (*B. empetrifolia*) and “michay de cordillera” (*B. serratodentata*). According to the CIE values, some of the most important exotic species were *Rosa rubiginosa*, *Prunus cerasus*, *Sambucus nigra*, *Prunus avium, Prunus domestica,* and finally *Rubus idaeus,* which is known throughout the world (Fig. 2a).

The PEFF in Cuyín Manzano belonged to eight botanical families. Rosaceae contributed most to the list (8 species), followed by Grosulariaceae (4 species) and Berberidaceae (3 species). However, the families with the highest CIE were Berberidaceae, Rosaceae and Elaeocarpaceae.

#### Local foods with PEFF

In Cuyín Manzano 44 local foods with PEFF were registered. Of these, 45% were consumed as fresh fruits. In general, this method of use implied consumption at the time of gathering, with no storage involved. Locals reported consuming these fruits when they were out in the countryside and felt hungry, the main reasons being that they like them a lot (such as the *Berberis* fruits), and they are “good for them”. To a lesser extent, the PEFF were lightly processed, through the addition of sugar or cream. Also frequently consumed were sweets (23%), including jams, jellies and syrups made from mature fruit.

Among other preparations (23%), were the alcoholic beverages, which in this case played a role as social drinks. The most frequently cited of these was the “guindado” made with the fruit of the exotic *Prunus cerasus,* which was served to visitors in the home. Non-alcoholic drinks represented 9% of this category, mainly in the form of infusions, such as those prepared with the fruit of *Rosa rubiginosa* and *Sambucus nigra,* but also in the form of juices.

Other forms of consumption were also mentioned, such as the addition of fruit to baking products in the home. Furthermore, the fruit tended to be used in the form of desserts, as in the case of stewed fruit, where fresh fruit such as plums were boiled in water and sugar. Consumption of dried fruit called “orejones” was also registered. It is worthy of note that the preparation of “chicha” from PEFF was not mentioned here as it was in the review.

#### Richness and use pattern (CIF) of functional PEFF

A total of 6 species (30%) were known to locals for their functional value (Table 4); however, the proportion of edible and/or functional species did not differ statistically (p > 0.05, binomial test). The functional species included 4 native and 2 exotic species. In contrast to the review, in Cuyín Manzano the CIF values were similar for native and exotic species (p > 0.05, Mann Whitney test).

**Table 4:** Functionals PEFF from Cuyín Manzano (Argentina)

Specie	CIF	UV	Ailments	Medicine and used part
*Rosa rubiginosa* L.	73	0,82	RS	Inf(f)
			DE	Oi(f)
*Sambucus nigra* L.	73	0,91	RS, U, OT	Inf(f)
			RS	De(f)
			RS, U	Sy(f)
*Aristotelia chilensis* (Molina) Stuntz	36	0,45	GI	F
			RS	Inf(f)
*Ribes magellanicum* Poir.	18	0,27	BL, DE	De (ro-pl)
*Berberis microphylla G.Forst.*	9	0,09	GI	F
*Ribes cucullatum* Hook. & Arn.	9	Nm	Nm	Nm

*RS* respiratory, *DE* dermatologic, *GI* gastrointestinal, *U* urinary, *OT* others, *In* infusion, *De* decoction, *F* fruit ingestion, *Sy* syrup, *Oi* ointment; *f* fruit, *ro* root, *pl* whole plant; *Nm* not mentioned

As shown in Table 4, the functional PEFF with highest CIF were the exotic *Rosa rubiginosa* and *Sambucus nigra*, followed by the native *Aristotelia chilensis*, *Ribes magellanicum*, *Berberis microphylla* and *Ribes cucullatum.*

The PEFF in Cuyín Manzano belonged to 5 botanical families: Grossularaciaceae (2 species), and Rosaceae, Berberidaceae, Elaeocarpaceae and Adoxaceae (1 species each). In terms of CIF, the families that stood out were: Adoxaceae (73%), Rosaceae (73%), and to a lesser extent, Elaeocarpaceae (36%).

The PEFF in Cuyín Manzano were used principally to treat respiratory, gastrointestinal and dermatological ailments (Table 4). *Sambucus nigra* was one of the most versatile species, followed by *Rosa rubiginosa*, *Aristotelia chilensis* and *Ribes magellanicum*, and finally, *Berberis microphylla*.

#### Medicines with PEFF

The inhabitants of Cuyín Manzano used 11 medicines prepared from PEFF to treat specific local ailments (Table 4). These species were mainly used in the form of infusions (33%), utilized generally to treat or prevent respiratory disease. One of the most frequently used infusions was sauco (elderberry) tea, which was drunk at night to “warm up the body”, and probably played a preventative role. This infusion was prepared by putting a spoonful of *Sambucus nigra* syrup in a cup, then adding boiling water. The syrup was also consumed alone, without dilution, mainly by children but also by adults suffering cold symptoms (25%). Mosqueta (rosehip) tea was used similarly as a recreational drink, to warm up the body and treat illness.

Fresh fruits were always consumed (17%) in the context of relieving gastrointestinal problems, and decoctions were also prepared (17%). The only external method of application was using creams (8%) made from *Rosa rubiginosa* fruits by women from a nearby community. The medicinal parts of functional PEFF are mainly the fruits (34%) (Table 4), and to a lesser extent, leaves (17%), the whole plant (17%), followed by flowers, seeds, bark and roots (8% each).

Finally, five of the medicines were also considered as food by locals: elderberry and/or rosehip infusions, the fresh fruits of maqui and michay, and elderberry syrup, which in total represent 11% of local foods.

## Discussion

The bibliographical analysis revealed a high number of native and exotic PEFF (73 species), providing an overall picture (spanning 70 years and an ample geographical range) that shows the study potential of all these Patagonian species.

The nutritional role of this plant diversity and the safety of their use is still unknown, but they presumably represent food security for locals. Food security is founded on three elements: the availability, access, and utilization of food, elements that were elucidated indirectly in both the bibliographical texts and the testimonies of Cuyin Manzano inhabitants. It is also important to consider that the conservation status of some of the native species (15 species, Table 1) is challenging, and must be solved by cultivation and/or sustainable management, given that their availability and access are threatened. The need for an integrated study of these aspects must be taken into account in future investigations.

Moreover, the field study showed that 20 PEFF on this list continue to be used. Although this rural community is probably experiencing a process of dietary transition from smallholder farming-based to industrialized food systems, as well as integration into the global food trade, PEFF continue to be part of their local heritage. Lifestyle changes probably affect food production, plant gathering and consumption, with consequences for the healthfulness of diets, but these species still have an appreciated role in home-produced foods. Further field research should be carried out in other Patagonian communities, using this tool to survey food security in order to investigate whether local people keep PEFF use alive in their food traditions. This aspect is of great importance given the processes of abandonment and loss of knowledge associated with plant use observed in Cuyín [50] and numerous other rural communities throughout the world [51].

Our cross-sectional approach coincides in the cultural importance of the three native species with highest consensus: *Berberis microphylla*, *Fragaria chiloensis* and *Aristotelia chilensis.*

The michay plant has attractive blue-violet berries with a flavor which is sweet, but sour, and is widely distributed in forest and the Patagonian steppe, therefore present also in the most arid zones. The native strawberry has strongly scented fruits, and has been used to obtain *Fragaria x annanasa*, the strawberry which is commercially cultivated throughout the world [56]. The berries of the maqui plant are acid to the taste, and have been designated a superfood due to their high content of anthocyanins [57, 58].

At present these species are being intensively studied as target species [19] and are attracting much attention for future innovation and development projects [59, 60]. The remainder of the list of PEFF may also be considered by policy makers and researchers in the planning of agricultural and phytochemical research action that will support sustainable diets and local food systems.

All this biodiversity is introduced into local gastronomy through a large variety of local foods. Remarkably, in both studies the use of fresh fruit is high (43% in the review and 45% in Cuyín Manzano). Traditional culinary habits could promote better use of nutrients, since a lower level of processing generally leads to greater availability of nutrients such as vitamin C [61]. In second place, the preparation of preserves and beverages from the fruit indicates strategies that favor preservation over time, storage, and the use of excess fruit, as found in other parts of the world [62, 63]. Preserves include some forms, such as syrups, which are of great interest due to the compounds that contribute phenolic acids, fiber, and soluble solids [64–66]. These plant preparations could be also highlighted, as local people could be skillfully managing the plant chemicals during food processing.

Patagonian communities have experimented extensively with the diversity of PEFF fruits in the preparation of fermented beverages, mainly in the form of “chicha”. This drink is made with fruit and seeds, which in many cases were chewed by the people before being stored for fermentation. This preparation holds great cultural and spiritual value, as it is drunk in traditional Mapuche festivities [67]. Nevertheless, in Cuyín Manzano preparation of these beverages with the PEFF berries was not identified. Although the medicinal use of these preparations was not documented, some studies associate moderate consumption of fermented beverages with prevention of cardiovascular diseases and cancer, due to their polyphenol and alcohol content [68]. This opens up a wide range of possibilities for the exploration of fermented beverages, taking into account ancient food traditions. These customs are considered key elements for development projects related to food sovereignty [69].

The PEFF heritage possesses a great richness of species whose functional value has been well taken advantage of, and which can therefore constitute alternative healthy or phytotherapeutic food. The 31 species taken from the bibliography and listed here are mainly native to Patagonia, and represent 31 opportunities for study, for which the consensus values can be a useful guide. In this sense, our findings are in accordance with Jennings et al. [70] as to functional species use. To understand the meaning of their functionality, contextualization of the food-plant spectrum based on both local beliefs and wider structural factors is needed. In our field study we found that dwellers consider that the fruit did them good. From the Mapuche worldview, their consumption of wild fruits implies nourishing the Earth’s energy (called in native Mapuzungum *“*afutum*”*), energy that cannot be transferred in any other way [71]. In other words, the classification of a species as functional is inseparably supported by both the local worldview and its chemical and biological attributes.

A whole universe of chemical compounds may be related to the health effects of the PEFF. The medicinal value of the species used as food has been explained in detail by authors such as Johns [72, 73], based on the presence of a wide range of phytoconstituents. Today we know that food plants have diverse constituents such as polyphenols, in addition to nutritional compounds. These molecules are recognized for their antioxidant properties; they are important radical scavengers through inhibition of prooxidant enzymes and restoration of antioxidant enzymes. However, new mechanisms of action have been proposed to explain their bioactivity, such as interaction at the level of the plasmatic membrane with proteins and phospholipids, and the regulation of signal transduction pathways [74, 75].

Of the PEFF, those which have berry-type fruits stand out as functional species, and are known worldwide as healthy foods. Berries contain vitamins, minerals and phenolic compounds, and are credited with antioxidant, antiaging, chemoprotective and chemotherapeutic, anti-inflammatory and neuroprotective properties [76, 77]. With a strong consensus between our two studies, the key species are: *A. chilensis*, *Ribes magellanicum*, *Ephedra ochreata* and *Berberis microphylla*. *A. chilensis* is the species with the highest number of described pharmacological properties, including the ability to attenuate pain and inflammation [74]. This activity matches the local ailment treatments described in our work. Little is known about *Ribes magellanicum*, but the antioxidant potential in vitro of the fruits found in Argentine-Chilean Patagonia has been demonstrated [14].

Unfortunately, *Ephedra ochreata* is one of the least-studied species in the region. Concerns about it eventual toxicity, should be carefully considered, since other *Ephedra* spp. have been demonstrated to exhibit toxicity (https://www.health.harvard.edu/staying-healthy/the-dangers-of-the-herb-ephedra). It must be used with great care, given that toxic effects have been reported for other species of *Ephedra*, such as cardiac and psychiatric disorder [78–80]. Future research should focus on *E. ochreata* in order to test its food safety.

The study of other species, such as *B. microphylla,* have begun only recently, revealing promising antioxidant content and antidiabetogenic activity according to in vitro and in vivo studies [81–83]. Again, more studies and further analysis is required to relate these chemical and biological findings to the health claims presented here.

Similar to the PEFF heritage, the use of functional species seems to be undergoing hybridization processes. This was evident in the rural community we worked in, where the CIF values were similar in native and exotic plants. These results show how locals have been using a combination of native and foreign species in edible and functional terms. Although this has been happening for a long time in the region, it seems to be more prevalent at the present time [84], probably due to changes in food procurement.

The use of exotic functional PEFF in our region may be interpreted as a reflection of the long, strong historical influence of European immigration on the use patterns of this area.

Various studies carried out in South America have shown that the introduction of Eurasian plants led to diversification in the use of medicinal plants [85], and many edible fruits form part of the pharmacopeias brought from Europe [86]. Shikov et al. [87] and Totelin [88] suggest that the use of edible fruits as medicine is due to the great influence of the food-medicine conceptions of Ancient Greece. However, the food-medicine interface had also been described previously, without the use of exotic plants, in Patagonian indigenous communities [89]. We suggest, therefore, that this food-medicine continuum may have been generated independently, and that the knowledge of indigenous and European communities converged at a later date.

Some of the exotic berries registered in this work have been extensively studied with regard to the biological activity that supports their local functional use. In the case of *Sambucus nigra,* there is in vitro evidence to support the effectiveness of its antibacterial and antiviral properties [76, 90]. Hawkins et al. [91] recently found that elderberry fruit can improve respiratory complaints associated with influenza, and so McCarty and DiNicolantonio [92] propose its use (in a dose of 600–1500 mg) for the treatment of COVID-19 infection. *Rosa canina*, a species closely related to *Rosa rubiginosa*, has been studied with regard to its use in treating skin ailments [93]. The dermatological use of *R. rubiginosa* and use of the other functional exotic species were also registered in our field work. This pattern shows how the berry repertoire grows with input from highly reputed exotic plants.

With respect to ailments, the review shows that more than 17 ailments have been treated with PEFF. In Cuyín Manzano this number is much lower (only five ailments) and is focused on primary health care issues. The broad scope of the review shows the potential of these species, even though the main ailments treated with PEFF are those most frequently treated by traditional medicine, such as digestive and respiratory complaints. Several studies have proposed that this redundancy gives flexibility to the regional herbal medicine, the use of *A. chilensis* and *S. nigra* fruits standing out as being the most versatile elements the population has within its reach [44].

The PEFF are also important sources of herbal medicines (54 in the review and 11 in Cuyín Manzano) since, as previously shown, different plant parts of the PEFF are used in an integral way. Infusions (and decoctions according to the bibliography) constitute the main method of medicinal use in the region, and this is also, within local foods, the main form of beverage in Cuyín Manzano. Recent research has revealed how infusions of exotic *Rosa rubiginosa* fruit can contribute compounds with antioxidant activity [94]. The same occurs with the infusion of exotic *Sambucus nigra* fruit, which was shown to contain considerable amounts of polyphenols and anthocyanins, comparable to an ethanolic extract [95]. Infusions are therefore, in general, good vehicles for phenolic compounds, and this is possibly the basis for their functional value in Patagonian cultures.

**Fig. 3:**
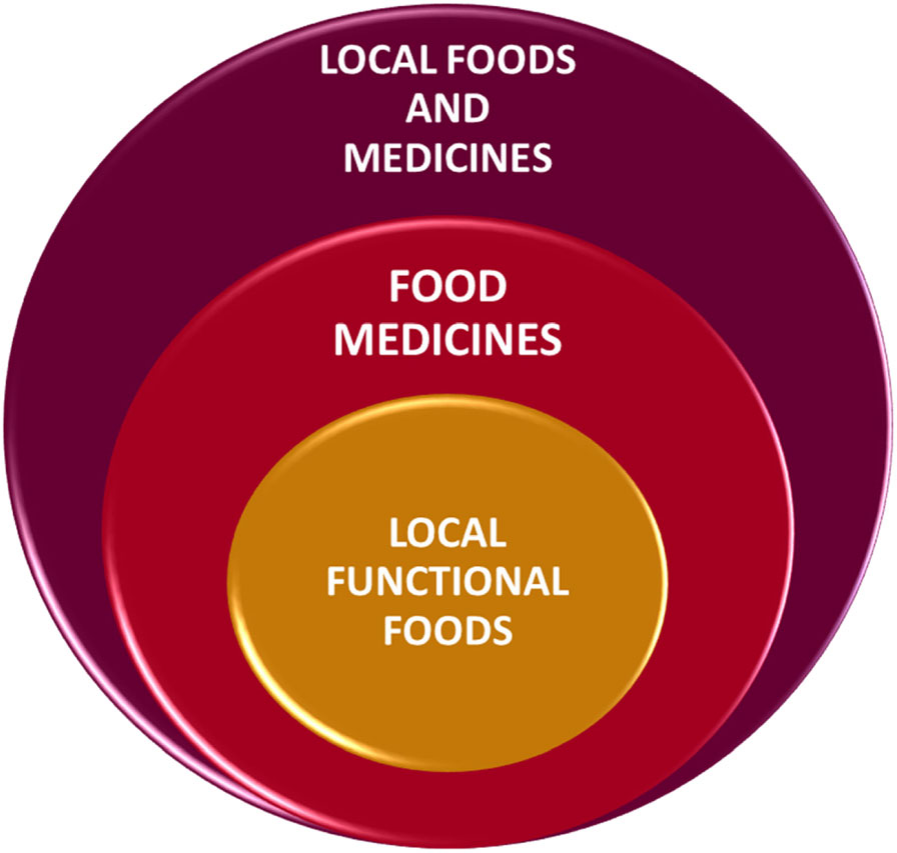
Conceptual framework of food-medicine interface

According to our analysis of the information gathered from the review, complemented by the fieldwork, we can reinterpret the comprehensiveness of the food-medicine interface in Patagonia, outlined in Fig. 3. In the review work we found a notable superposition of use (edible and medicinal) for single native or exotic species (given by the sum of citations of different authors and different study sites). However, in the analysis of each one of the studies included in the review and the fieldwork, we observed that the Patagonian communities have not only experimented with native and exotic plants and discovered their medicinal properties, but they have also developed diverse forms of preparation. These make up a wide range of methods of use, but also form part of local food tradition, which considers plants from the perspective of functional, complementary logic, for their general health in their daily lives, local foods and medicines. Within this group there are forms of preparation that prove effective as food and medicine (superposition of use forms depending on context), known in the literature as food medicines or healthy foods [62].

Within this group we find the subgroup of the local functional foods. As shown in our field study, this is the case of *Rosa rubiginosa* and *Sambucus nigra*, two exotic berries that are used in the form of infusions and indistinguishable as food or medicine. They are used as a preventative food, which in nutrition is known as a functional food [7]. These infusions seem to be important local functional foods, given that these water solutions are good vehicles for active compounds found in plants, such as polyphenols [96, 97] .

Finally, the functionality attributed by a culture to its native and exotic species includes superposition at different levels, and may be described in theoretical terms as different categories. However, most important is that they are principally based on the use of the entire plant. In the case of Patagonian native and exotic plants with edible fruit, the people seek food, healing, and also prevention (“it warms up the body”, “it does you good”) through the use of infusions and the ingestion of fresh fruit. Previous studies have shown the potential of some native PEFF, due to their polyphenol content, but little has been studied on the teas prepared with them. More integrative research is required into this use pattern of native and exotic species, their local foods and their potential as local functional foods.

## Conclusions

Argentine Patagonia holds great richness of native and exotic PEFF and the cultures who have lived on these lands have experimented with them as a source of local foods and medicines, generating a food-medicine continuum. The living heritage of Patagonian PEFF appears to have undergone hybridization processes, such that exotic cosmopolitan species play an increasingly significant role.

Ethnobotanical research can contribute solutions to health problems worldwide by surveying food security and investigating whether local people maintain PEFF use alive in their food traditions. This should be carried out with locals in a collaborative way, and this synergic effort could provide ideas and opportunities for the sustainable and multipurpose use of plants. The development of functional local foods in any culture could offer contemporary strategies for preserving ecological and cultural diversity, which includes both the tangible and the intangible cultural heritage.

## Data Availability

The datasets used and/or analyzed during the current study are available from the corresponding author on request.

## Abbreviations

PEFF: Plant with edible fleshy fruit
CIE: Edible consensus index
CIF: Functional consensus index
UV: Use Value

## References

[1] GBD 2017 DC (2019). Health effects of dietary risks in 195 countries, 1990–2017: a systematic analysis for the Global Burden of Disease Study 2017. Lancet.

[2] Liu RH (2004). Potential synergy of phytochemicals in cancer prevention: mechanism of action. J Nutr.

[3] Seeram NP, Adams LS, Henning SM, Niu Y, Zhang Y, Nair MG (2005). In vitro antiproliferative, apoptotic and antioxidant activities of punicalagin, ellagic acid and a total pomegranate tannin extract are enhanced in combination with other polyphenols as found in pomegranate juice. J Nutr Biochem.

[4] Wang S, Melnyk JP, Tsao R, Marcone MF (2011). How natural dietary antioxidants in fruits, vegetables and legumes promote vascular health. Food Res Int Elsevier Ltd.

[5] Forouhi NG, Unwin N (2019). Global diet and health: old questions, fresh evidence, and new horizons. Lancet.

[6] Howlett J, Aggett P, Walker R, Madsen C (2008). Functional foods from science to health and claims.

[7] Pieroni A, Quave CL, Pieroni A, Price L (2006). Functional foods or food medicines? On the consumption of wild plant among Albanians and southern Italians in Lucania. Eating and healing: traditional food as medicine.

[8] Heinrich M, Nebel S, Leonti M, Rivera D, Obón C (2006). Local Food-Nutraceuticals: Bridging the gap between local knowledge and global needs. In: Heinrich M, Müller WE, Galli C, editors. Local mediterranean food plants and nutraceuticals.

[9] Etkin NL, Ross PJ (1982). Food as medicine and medicine as food: an adaptative framework for the interperetation of plant utilization among the hausa of northern Nigeria. Soc Sci Med.

[10] Arena ME, Radice S (2014). Shoot growth and development of Berberis buxifolia lam. In Tierra del Fuego (Patagonia). Sci Hortic (Amsterdam). Elsevier.

[11] Arena ME, Coronel LJ (2011). Fruit growth and chemical properties of Ribes magellanicum “parrilla”. Sci Hortic (Amsterdam). Elsevier B.V.

[12] Cheel J, Theoduloz C, Rodríguez JA, Caligari PDS, Schmeda-Hirschmann G (2007). Free radical scavenging activity and phenolic content in achenes and thalamus from Fragaria chiloensis ssp. chiloensis, F. vesca and F. x ananassa cv. Chandler. Food Chem.

[13] Escribano-Bailón MT, Alcalde-Eon C, Muñoz O, Rivas-Gonzalo JC, Santos-Buelga C (2006). Anthocyanins in berries of Maqui (Aristotelia chilensis (Mol.) Stuntz). Phytochem Anal.

[14] Jiménez-Aspee F, Thomas-Valdés S, Schulz A, Ladio A, Theoduloz C, Schmeda-Hirschmann G (2015). Antioxidant activity and phenolic profiles of the wild currant Ribes magellanicum from Chilean and Argentinean Patagonia. Food Sci Nutr.

[15] Brauch JE, Buchweitz M, Schweiggert RM, Carle R (2016). Detailed analyses of fresh and dried maqui (Aristotelia chilensis (Mol.) Stuntz) berries and juice. Food Chem. Elsevier Ltd.

[16] Damascos MA, Arribere M, Svriz M, Bran D (2008). Fruit mineral contents of six wild species of the north Andean Patagonia, Argentina. Biol Trace Elem Res.

[17] Alvarado JL, Leschot A, Olivera-Nappa Á, Salgado AM, Rioseco H, Lyon C (2016). Delphinidin-rich maqui berry extract (Delphinol®) lowers fasting and postprandial glycemia and insulinemia in prediabetic individuals during oral glucose tolerance tests. Biomed Res Int.

[18] Jiménez-Aspée F, Theoduloz C, Ladio A, Schmeda-Hirschmann G (2015). Patagonian Ribes and Rubus: native fruits with nutraceutical potencial. XIth International Rubus and Ribes Symposium, NC State University Plants for Human Health Institute. Asheville, North Carolina, USA.

[19] Schmeda-Hirschmann G, Jiménez-Aspee F, Theoduloz C, Ladio A (2019). Patagonian berries as native food and medicine. J Ethnopharmacol. Elsevier Ireland Ltd.

[20] Chamorro MF, Ladio AH, Molares S, Martínez J, Muñoz-Acevedo A, Rai M (2018). Patagonian berries: an ethnobotanical approach to exploration of their nutraceutical potential. Ethnobotany: local knowledge and traditions.

[21] Ladio AH, Lozada M (2001). Non-timber forest product use in two human populations from NW Patagonia: A quantitative approach. Human Ecol.

[22] Ladio AH, Rapoport E (1999). El uso de plantas silvestres comestibles en una población suburbana del noroeste de la Patagonia. Parodiana..

[23] Ladio AH (2011). Traditional knowledge of edible wild native and exotic plants in the context of cultural change in human populations of arid Patagonai. Bioremediation, Biodivers Bioavailab.

[24] Ladio AH (2017). Ethnobiology and research on global environmental change: what distinctive contribution can we make?. Ethnobiol Conserv..

[25] Eyssartier C, Ladio AH, Lozada M (2015). Horticultural practice and germplasm conservation: a case study in a rural population of the Patagonian steppe. Food Secur.

[26] Ladio AH, Albuquerque UP (2014). The concept of hybridization and its contribution to urban ethnobiology. Ethnobiol Conserv.

[27] Hurrell JA, Ulibarri EA, Delucchi G, Pochettino ML, Hurrell JA (2010). Frutas: frescas, secas y preservadas.

[28] Ladio AH, Molares S, Raffaele E, de Torres CM, Morales C, Kitzberger T (2014). El paisaje patagónico y su gente. Ecología e historia natural de la Patagonia Andina: un cuarto de siglo de investigación en biogeografía, ecología y conservación.

[29] Raffaele E, de Torres CM, Morales C, Kitzberger T (2014). Ecología e historia natural de la Patagonia Andina: un cuarto de siglo de investigación en biogeografía, ecología y conservación.

[30] Medeiros MFT (2009). Etnobotânica histórica: princípios e procedimentos. Ecologia e Etnobotânica Aplicada.

[31] Ezcurra C, Brion C (2005). Plantas del Nahuel Huapi. Catálogo de la flora vascular del Parque Nacional Nahuel Huapi, Argentina.

[32] Correa MN (1998). Flora Patagónica. Introducción.

[33] Hajduk A, Albornoz AM, Lezcano MJ (2008). Arqueología del Parque Nacional Nahuel Huapi (Provincia de Río Negro y Neuquén). Las ocupaciones tempranas de la transición Pleistoceno-Holoceno al Holoceno Medio en el área boscosa-lacustre 1 . Cuartas Jornadas de Historia de la Patagonia. Bariloche.

[34] Lozada M, Ladio A, Weigandt M (2006). Cultural transmission of Ethnobotanical knowledge in a rural Community of Northwestern Patagonia, Argentina. Econ Bot.

[35] Ochoa JJ, Ladio AH (2015). Plantas silvestres con órganos subterráneos comestibles: transmisión cultural sobre recursos subutilizados en la Patagonia (Argentina). Bol Latinoam y del Caribe Plantas Med y Aromat..

[36] Ochoa JJ, Ladio AH (2014). Ethnoecology of Oxalis adenophylla Gillies ex hook. & Arn. J Ethnopharmacol. Elsevier.

[37] Molares S, Ladio A (2009). Chemosensory perception and medicinal plants for digestive ailments in a Mapuche community in NW Patagonia, Argentina. J Ethnopharmacol.

[38] Heinrich M, Lardos A, Leonti M, Weckerle C, Willcox M, Applequist W (2018). Best practice in research: consensus statement on Ethnopharmacological field studies – ConSEFS. J Ethnopharmacol. Elsevier Ireland Ltd.

[39] Albuquerque UP (2010). Paiva de Lucena RF, Cruz da Cunha LVF, editors. Métodos e técnicas na pesquisa Etnobiológica y Etnoecológic.

[40] Alexiades MN (1996). Selected guidelines for Ethnobotanical Reserach: a field manual.

[41] Albuquerque UP, Fernandes Cruz da Cunha LV, Paiva de Lucena RF, Nóbrega Alves RR, Albuquerque UP (2014). Methods and Techniques in Ethnobiology and Ethnoecology.

[42] Conover WJ (1971). Practical nonparametric statics.

[43] Eyssartier C, Ladio AH, Lozada M (2008). Cultural transmission of traditional knowledge in two populations of North-Western Patagonia. J Ethnobiol Ethnomed.

[44] Molares S, Ladio A (2009). Ethnobotanical review of the Mapuche medicinal flora: use patterns on a regional scale. J Ethnopharmacol.

[45] Cornara L, La Rocca A, Terrizzano L, Dente F, Mariotti MG (2014). Ethnobotanical and phytomedical knowledge in the North-Western Ligurian Alps. J Ethnopharmacol Elsevier.

[46] Tardío J, Pardo-de-Santayana M (2008). Cultural importance indices: a comparative analysis based on the useful wild plants of southern Cantabria (northern Spain)1. Econ Bot.

[47] Phillips O, Gentry AH (1993). The useful plants of Tambopata, Peru: I. statistical hypotheses tests with a new quantitative technique. Econ Bot.

[48] Tardío J, Pardo-De-Santayana M (2008). Cultural importance indices: a comparative analysis based on the useful wild plants of southern Cantabria (northern Spain). Econ Bot.

[49] Albuquerque UP, Lucena RFP, Monteiro JM, Florentino ATN, Almeida C (2006). de FCBR. Evaluating two quantitative Ethnobotanical techniques. Ethnobot Res Appl.

[50] Correa MN (1971). Flora Patagónica. Compositae.

[51] Correa MN (1984). Flora Patagónica. Dicotiledóneas, dialipétalas (Salicaceae a Cruciferae).

[52] Correa MN (1988). Flora Patagónica. Dicotiledóneas, dialipétalas (Oxalidaceae a Cornaceae).

[53] Landrum LR (2007). Revision of Berberis (Berberidaceae) in Chile and adjacent southern Argentina. Ann of the Missouri Bot Gard.

[54] Ragonese AE, Martinez CR (1947). Plantas indígenas de la Argentina con Frutos o Semillas comestibles. Rev Investig Agrícola.

[55] Martinez CR. Breve panorama de las plantas utilizadas por los indios de Patagonia y Tierra del Fuego. Supl Antropológico. 1982;XVII(1):61–97.

[56] Finn CE, Retamales JB, Lobos GA, Hancock JF (2013). The chilean strawberry (Fragaria chiloensis): over 1000 years of domestication. HortScience..

[57] Brauch JE (2016). Underutilized Fruits and Vegetables as Potential Novel Pigment Sources. Handbook on Natural Pigments in Food and Beverages: Industrial Applications for Improving Food Color. Elsevier Ltd.

[58] Ruiz A, Hermosín-Gutiérrez I, Mardones C, Vergara C, Herlitz E, Vega M (2010). Polyphenols and antioxidant activity of calafate (Berberis microphylla) fruits and other native berries from southern Chile. J Agric Food Chem.

[59] Pino MT, Leod CM, Ojeda A, Zamora O, Saavedra J (2017). Characterization and clonal selection of Berberis microphylla G. Forst in the Chilean Patagonia region for natural colorant purposes. Acta Hortic.

[60] Vogel H, Peñailillo P, Doll U, Contreras G, Catenacci G, González B (2014). Maqui (Aristotelia chilensis): Morpho-phenological characterization to design high-yielding cultivation techniques. J Appl Res Med Aromat Plants.

[61] Shahidi F (2009). Nutraceuticals and functional foods: whole versus processed foods. Trends Food Sci Technol Elsevier Ltd.

[62] Alarcón R, Pardo-De-Santayana M, Priestley C, Morales R, Heinrich M (2015). Medicinal and local food plants in the south of Alava (Basque Country, Spain). J Ethnopharmacol.

[63] Pieroni A, Sõukand R (2018). Forest as stronghold of local ecological practice: currently used wild food plants in Polesia, Northern Ukraine. Econ Bot.

[64] Abbès F, Kchaou W, Blecker C, Ongena M, Lognay G, Attia H (2013). Effect of processing conditions on phenolic compounds and antioxidant properties of date syru. Ind Crops Prod. Elsevier B.V.

[65] Quispe C, Petroll K, Theoduloz C, Schmeda-Hirschmann G (2014). Antioxidant effect and characterization of South American Prosopis pods syrup. Food Res Int. Elsevier B.V.

[66] Seglina D, Karklina D, Ruisa S, Krasnova I (2006). The effect of processing on the composition of sea buckthorn juice. J Fruit Ornam Plant Res.

[67] Pardo O, Pizarro JL (2005). La Chicha en el Chile Precolombino. First.

[68] Arranz S, Chiva-Blanch G, Valderas-Martínez P, Medina-Remón A, Lamuela-Raventós RM, Estruch R (2012). Wine, beer, alcohol and polyphenols on cardiovascular disease and cancer. Nutrients..

[69] Sõukand R, Pieroni A, Biró M, Dénes A, Dogan Y, Hajdari A (2015). An ethnobotanical perspective on traditional fermented plant foods and beverages in Eastern Europe. J Ethnopharmacol.

[70] Jennings HM, Merrell J, Thompson JL, Heinrich M (2015). Food or medicine? The food–medicine interface in households in Sylhet. J Ethnopharmacol. Elsevier.

[71] Ladio AH, Molares S, Casas A, Torres-Guevara J, Parra Rondinel F (2017). Etnoconservacionismo y prácticas locales en Patagonia: avances y perspectivas. Domesticación en el continente americano: Investigación para el manejo sustentable de recursos genéticos en el Nuevo Mundo.

[72] Johns T (1990). With bitter herbs they shall eat it: chemical ecology and the origins of human diet and medicine.

[73] Johns T (1999). The chemical ecology of human ingestive behaviors. Annu Rev Anthropol.

[74] Kim HS, Quon MJ, Kim JA (2014). New insights into the mechanisms of polyphenols beyond antioxidant properties; lessons from the green tea polyphenol, epigallocatechin 3-gallate. Redox Biol. Elsevier.

[75] Vauzour D, Rodriguez-Mateos A, Corona G, Oruna-Concha MJ, Spencer JPE (2010). Polyphenols and human health: prevention of disease and mechanisms of action. Nutrients..

[76] Nile SH, Park SW (2014). Edible berries: Bioactive components and their effect on human health. Nutrition. Elsevier Inc.

[77] Paredes-López O, Cervantes-Ceja ML, Vigna-Pérez M, Hernández-Pérez T (2010). Berries: improving human health and healthy aging, and promoting quality life-a review. Plant Foods Hum Nutr.

[78] Shekelle PG, Hardy ML, Morton SC, Maglione M, Mojica WA, Suttorp MJ (2003). Efficacy and safety of Ephedra and ephedrine for weight loss and athletic performance: a meta-analysis. J Am Med Assoc.

[79] FDA (1994). Alerts, Advisories & Safety Information.

[80] Haller CA, Benowitz NL (2000). Adverse cardiovascular and central nervous system events associated with dietary supplements containing ephedra alkaloids. N Engl J Med.

[81] Soto-Covasich J, Reyes-Farias M, Torres RF, Vasquez K, Duarte L, Quezada J (2020). A polyphenol-rich Calafate (Berberis microphylla) extract rescues glucose tolerance in mice fed with cafeteria diet. J Funct Foods Elsevier.

[82] Chamorro MF, Reiner G, Theoduloz C, Ladio A, Schmeda-hirschmann G, Gómez-Alonso S (2019). Berberis species and wild strawberry from the Argentinean Patagonia. Molecules..

[83] Garcia-Diaz DF, Jimenez P, Reyes-Farias M, Soto-Covasich J, AGV C (2019). A Review of the Potential of Chilean Native Berries in the Treatment of Obesity and its Related Features. Plant Foods Hum Nutr.

[84] Molares S, Ladio AH (2015). Complejos vegetales comestibles y medicinales en la Patagonia Argentina : sus componentes y posibles procesos asociados. Bol Latinoam y del Caribe Plantas Med y Aromat.

[85] Hanazaki N, Peroni N, Begossi A. Edible and Healing Plants in the Ethnobotany of Native Inhabitants of the Amazon and Atlantic Forest Areas of Brazil. In: Pieroni A, Price LL, editors. Eating and Healing - traditional food as medicine. New York: Haworth Press; 2006. p. 251–71.

[86] Kujawska M, Pieroni A (2015). Plants used as food and medicine by polish migrants in Misionesl, Argentina. Ecol Food Nutr.

[87] Shikov A, Tsitsilin AN, Pozharitskaya O, Makarov VG, Heinrich M (2017). Traditional and Current Food Use of Wild Plants Listed in the Russian Pharmacopoeia. Front Pharmacol.

[88] Totelin L (2015). When foods become remedies in ancient Greece: the curious case of garlic and other substances. J Ethnopharmacol. Elsevier.

[89] Ladio A, Pieroni A, Price L (2006). Gathering of wild plant foods with medicinal use in a Mapuche community of Northwest Patagonia. Eating and healing: traditional food as medicine.

[90] Młynarczyk K, Walkowiak-Tomczak D, Łysiak GP (2018). Bioactive properties of Sambucus nigra L. as a functional ingredient for food and pharmaceutical industry. J Funct Foods.

[91] Hawkins J, Baker C, Cherry L, Dunne E (2019). Black elderberry (Sambucus nigra) supplementation effectively treats upper respiratory symptoms: a meta-analysis of randomized, controlled clinical trials. Complement Ther Med Elsevier Ltd.

[92] McCarty MF, DiNicolantonio JJ. Nutraceuticals have potential for boosting the type 1 interferon response to RNA viruses including influenza and coronavirus. Prog Cardiovasc Dis. Elsevier Inc. 2020. p. 79–81. Preprint at https://www.ncbi.nlm.nih.gov/pmc/articles/PMC7130854/. 10.1016/j.pcad.2020.02.007 PMC7130854 32061635

[93] Chrubasik C, Roufogalis BD, Ulf M-L, Chrubasik S (2008). A systematic review on the Rosa canina effect and efficacy profile. Phyther Res.

[94] Jiménez-López J, Ruiz-Medina A, Ortega-Barrales P, Llorent-Martínez EJ (2017). Rosa rubiginosa and Fraxinus oxycarpa herbal teas: characterization of phytochemical profiles by liquid chromatography-mass spectrometry, and evaluation of the antioxidant activity. New J Chem.

[95] Duymuş HG, Göger F, Başer KHC (2014). In vitro antioxidant properties and anthocyanin compositions of elderberry extracts. Food Chem.

[96] Atoui AK, Mansouri A, Boskou G, Kefalas P (2005). Tea and herbal infusions: their antioxidant activity and phenolic profile. Food Chem.

[97] Carabajal MPA, Isla MI, Zampini IC (2017). Evaluation of antioxidant and antimutagenic activity of herbal teas from native plants used in traditional medicine in Argentina. South African J Bot SAAB.

